# Industrially Employed Mothers

**Published:** 1921-05-28

**Authors:** 


The Workers' Newspaper of Administrative Medicine and Institutional Life, Administration, National Insurance and Health.

No. 1825, Vol. LXX. SATURDAY, MAY 28, 1921. PRICE TWOPENCE

### INDUSTRIALLY EMPLOYED MOTHERS.

It is, we think, extremely improbable that the Ministry of Health will take action on the memorandum prepared by Sir Alfred Watson, the Government Actuary, in the Washington Draft Convention concerning the employment of women before and after childbirth. The Convention provides that no woman (whether married or single) shall be permitted to work during the six weeks following her confinement in any commercial or industrial undertaking, and that she shall have the right to leave her work upon the production of a medical certificate stating that her confinement will probably take place within six weeks. While a woman is absent from her work in these circumstances she is to be paid benefits sufficient for the full and healthy maintenance of herself and her child, and is in addition to receive free attendance by a doctor or certified midwife. The number of women to whom such a scheme would apply in a single year in this country is about 120,000. With a benefit fixed at no more than twenty shillings per week the cost for each woman would be £14, including £2 for medical attendance, and it is estimated that the general cost would be about £1,700,000 a year. (These figures appear to us, however, to have no real value when considered in close relation to the problems raised. For instance, it is conditional on the assumption that the benefits will not draw into the labour market a large number of married women who otherwise would never have sought industrial employment, with unpleasant economic consequences. There is also the question whether the new benefit would cause an increase of illegitimacy, and we fail to see how in practice the Government could long resist the demand for an extension of the benefits to mothers of every social class, irrespective of any question of employment. If the benefits were universal at £14 for each birth the cost would be considerably over £15,000,000 a year. Quite apart from the disinclination of the public at such a time as the present to spend that immense sum annually in addition to our growing commitments in all directions, and quite apart from the attitude of insured persons to the proposals, if the financial burden (as suggested) falls on them in the form of greatly increased insurance contributions, there is as yet no consensus of expert opinion as to what constitutes the best solution of the problem of child-bearing women in industry. It is an undoubted fact that in some occupations it is not only not harmful, but positively beneficial that a pregnant woman shall have light, regular work to do, which takes the place of mild exercise. Otherwise she might take no exercise at all, or if she stayed at home upon compulsion the danger is that she would jeopardise her health far more seriously by over-exertion in domestic duties than by carefully regulated work in the factory. The question bristles with difficulties, and we hope the Government will approach it with much caution.

